# Prevalence and Prognostic Impact of Malnutrition in Critical Patients With Acute Myocardial Infarction: Results From Chinese CIN Cohort and American MIMIC-III Database

**DOI:** 10.3389/fnut.2022.890199

**Published:** 2022-06-15

**Authors:** Jin Lu, Zhidong Huang, Junjie Wang, Xiaoli Zhao, Yanfang Yang, Bo Wu, Yu Kang, Jiaming Xiu, Jiabin Tu, Yuxiong Pan, Weihua Chen, Kunming Bao, Liling Chen, Jin Liu, Yong Liu, Shiqun Chen, Yong Fang, Kaihong Chen

**Affiliations:** ^1^Department of Cardiology, Longyan First Affiliated Hospital of Fujian Medical University, Longyan, China; ^2^Department of Cardiology, Guangdong Provincial Key Laboratory of Coronary Heart Disease Prevention, Guangdong Cardiovascular Institute, Guangdong Provincial People's Hospital, Guangdong Academy of Medical Sciences, Guangzhou, China; ^3^Department of Cardiology, The Third Affiliated Hospital of Sun Yat-sen University, Guangzhou, China

**Keywords:** malnutrition, critical acute myocardial infarction, all-cause mortality, Controlling Nutritional Status (CONUT) score, MIMIC-III database

## Abstract

**Background:**

Malnutrition is associated with poor prognosis in patients with acute myocardial infarction (AMI). However, the prognostic impact of malnutrition in critical patients with AMI has not been well addressed.

**Methods:**

We analyzed two critical AMI cohorts from Cardiorenal ImprovemeNt (CIN) in China and Medical Information Mark for Intensive Care-III (MIMIC-III) in the United States. The primary outcome was all-cause mortality. Cox proportional hazards models were constructed to examine the risk of malnutrition for mortality in critical patients with AMI.

**Results:**

There were 2,075 critical patients with AMI (mean age, 62.5 ± 12.3 years, 20.00% were female) from the CIN cohort and 887 critical patients with AMI (mean age, 70.1 ± 12.9 years, 37.43% were female) from MIMIC-III included in this study. Based on the Controlling Nutritional Status (CONUT) score, of the Chinese patients with AMI, the prevalence was 47.5, 28.3, and 3.5% for mild, moderate, and severe malnutrition, respectively. The percentage of mild, moderate, and severe malnutrition was 41.60, 30.55, and 7.32% in the MIMIC-III cohort, respectively. Controlling for confounders, worse nutritional state was significantly associated with increased risk for all-cause mortality [an adjusted hazard ratio for mild, moderate, and severe malnutrition, respectively, 1.10 (95% confidence interval (CI): 0.76–1.59), 1.49 (95% CI: 1.02–2.19), and 1.70 (95% CI: 1.00–2.88) in the CIN cohort and 1.41 (95% CI: 0.95–2.09), 1.97 (95% CI: 1.32–2.95), and 2.70 (95% CI: 1.67–4.37) in the MIMIC-III cohort].

**Conclusion:**

Malnutrition was independently associated with an increased risk of all-cause mortality in critical patients with AMI after full adjustments. Further trials are needed to prospectively evaluate the efficacy of nutritional interventions in critical patients with AMI.

## Introduction

Although the treatment with early reperfusion and pharmacological therapy has contributed to improved outcomes in patients with acute myocardial infarction (AMI) (1–3), the mortality remains high when complications such as cardiogenic shock, cardiac rupture, and in-hospital cardiac arrest occur in patients with AMI in coronary care unit (CCU) (4–6). Therefore, understanding more modifiable clinical variables to identify high-risk patients can optimize medical management.

Malnutrition has been identified as an independent predictor of unfavorable prognosis for cardiovascular diseases based on different nutritional assessment tools (7, 8). Therefore, the Controlling Nutritional Status (CONUT) score, including serum albumin, total cholesterol, and lymphocytes, is a simple and efficient tool to assess the nutritional status (9). In previous studies, the association has been verified between malnutrition and increased risk of mortality using the CONUT score in general AMI populations (10, 11). However, it remains unclear whether this association exists in critical patients with AMI with more severe pathophysiological conditions.

Therefore, the aim of the present study is to report the prevalence and prognostic consequences of nutritional status in critical patients with AMI from the Chinese CIN cohort and the American MIMIC-III database.

## Methods

### Data Sources

#### Chinese Cohort

Cardiorenal ImprovemeNt-database (CIN, ClinicalTrials.gov NCT04407936) is a real-world observational study focused on the improvement of cardiorenal complications of coronary intervention. The cohort mainly included patients undergoing coronary angiography (CAG) or percutaneous coronary intervention (PCI) from the large tertiary hospital between 2007 and 2018 in Guangdong Provincial People's Hospital, Guangdong, China.

#### American Cohort

MIMIC-III is a large, freely available database, comprising de-identified health-related data associated with over forty thousand patients who stayed in critical care units of the Beth Israel Deaconess Medical Center between 2001 and 2012. The database encompasses a diverse and very large population of Intensive Care Unit (ICU) patients; and it contains highly granular data, including vital signs, laboratory results, and medications. To access the database, we completed the National Institutes of Health's web-based course Protecting Human Research Participants.

### Study Population

**Figure 1:**
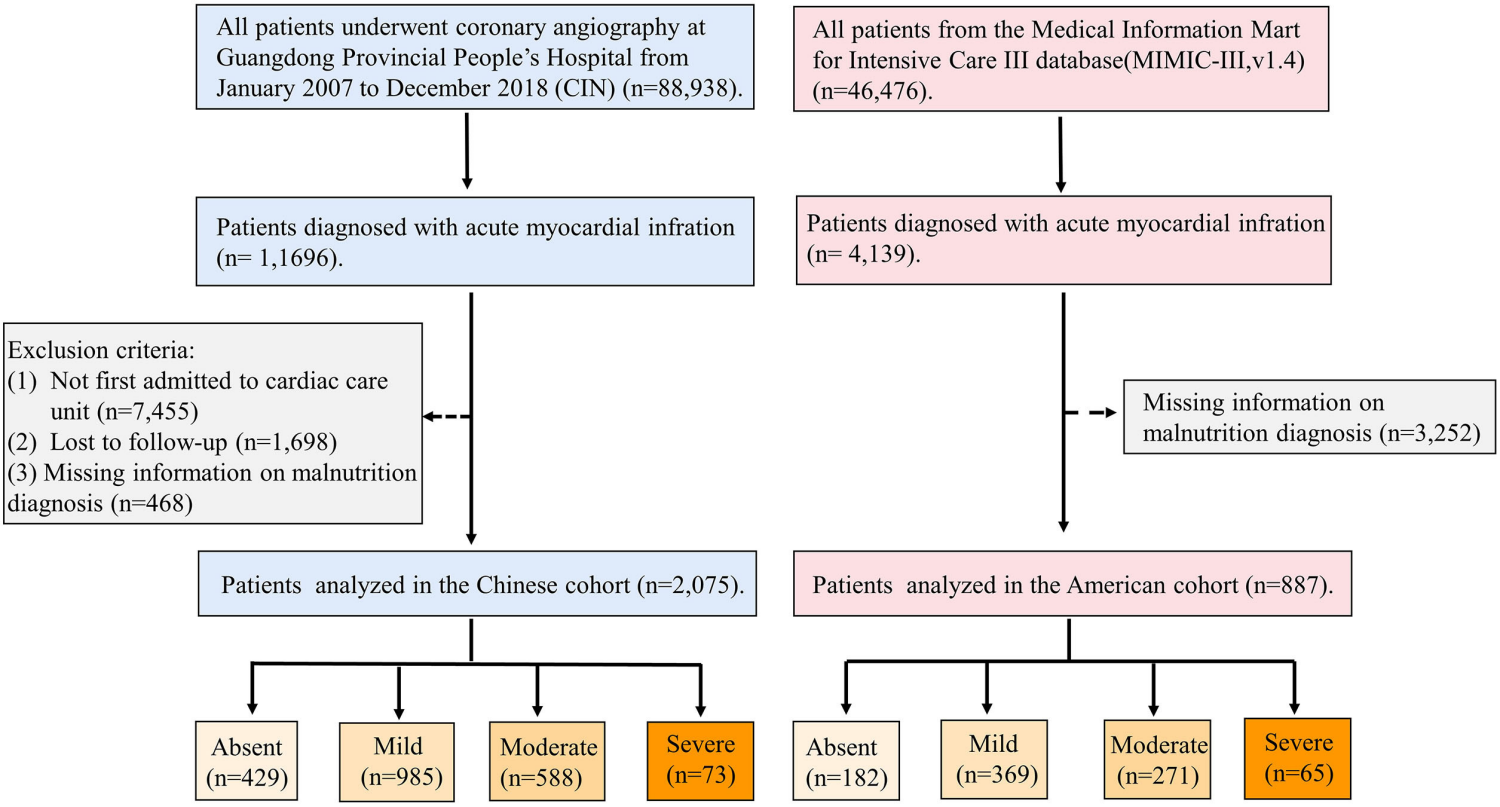
A patient flow diagram of Chinese CIN and American MIMIC-III databases.

This study enrolled patients with AMI who had been admitted to CCU. The patients with missing information on malnutrition diagnosis, cardiac and renal functions, and follow-up information were excluded. Eventually, 4,448 patients were included in the CIN, and 887 patients were included in the MIMIC-III (Figure 1). All traceable personal identifiers were removed from the analytic dataset to protect patients' privacy. The study was approved by the local ethics committee and was performed according to the Declaration of Helsinki.

### Nutritional Status

In both cohorts, malnutrition was screened using the CONUT score. The CONUT score of 0 to 1 is considered normal; scores of 2–4, 5–8, and 9–12 reflect mild, moderate, and severe malnutrition, respectively (9). A diagnosis of high-risk malnutrition was made if the COUNT score was >4 (Supplementary Table 1).

### Data Collection

The data of CIN were extracted from the electronic clinical management records system of the Guangdong Provincial People's Hospital. We had access to all primary and secondary care records. The CONUT score was determined by serum albumin, total cholesterol, and lymphocyte count collected in the early morning after overnight fasting at admission. The baseline information included demographic characteristics, coexisting conditions, laboratory examinations, and medications. Acute myocardial infarction (AMI), hypertension, diabetes, and stroke were defined using ICD-10 codes. The follow-up information was matched from the Guangdong Provincial Public Security based on the electronic Clinical Management System of the Guangdong Provincial People's Hospital records.

The MIMIC-III database includes information from 2002 to 2011. Hourly physiologic readings from bedside monitors, validated by CCU nurses, were recorded. The database also contains records of demographics, laboratory results, and other clinical variables. International Classification of Diseases, Ninth Revision (ICD-9) codes was also documented for specific diseases by hospital staff on patients' discharge. This information is extracted from the database based on structure query language (SQL). The follow-up started from the date of admission and ended at death.

### Endpoint and Clinical Definition

The primary endpoint was all-cause mortality. Anemia was defined as a hematocrit ≤ 39% (male) or ≤36% (female). Congestive heart failure (CHF) was defined as New York Heart Association (NYHA) class > 2 or Killip class > 1.

### Statistical Analysis

Continuous variables with baseline characteristics conforming to the normal distribution are presented as mean ± SDs, and the others not conforming to the normal distribution are presented as quartiles [median (25%, 75%)] by the Kolmogorov-Smirnov test. In addition, baseline characteristics are presented as proportions for categorical variables. The differences in baseline characteristics between groups were compared using the Student *t*-test for continuous variables and chi-square tests for categorical variables. Percent standardized differences (standardized differences ^*^100) were also provided. Time-to-event data among groups are presented graphically using Kaplan–Meier curves and compared by the log-rank test. Multivariable Cox regression models were used to examine the relationship between malnutrition and mortality. Model 1 was unadjusted. Model 2 was adjusted for age and gender. Model 3 was adjusted for age, gender, S-T segment elevation myocardial infarction (STEMI), chronic kidney disease, CHF, PCI, anemia, stroke, hypertension, diabetes, sodium ion, and potassium ion. We used propensity-matched analysis (PSM) and inverse probability weighting (IPW) adjustment to further explore the association between malnutrition and all-cause mortality. Presented tests were 2-tailed for all, and the *p*-value of < 0.05 was considered statistically significant. All statistical analyses were performed using R (ver. 4.0.3).

## Results

There were 2,075 critical patients with AMI (mean age, 62.5 ± 12.3 years, 20.00% were female) from the CIN cohort and 887 critical patients with AMI (mean age, 70.1 ± 12.9 years, 37.43% were female) from MIMIC-III included in this study. Based on the CONUT score, of the Chinese patients with AMI, the prevalence of all levels of malnutrition was 79.3% with mild, moderate, and severe malnutrition, being 47.5, 28.3, and 3.5%, respectively. The percentage of patients with malnutrition was 79.47% in the MIMIC-III cohort according to the CONUT score. The percentage of mild, moderate, and severe malnutrition was 41.60, 30.55, and 7.32% in the MIMIC-III cohort, respectively.

In both cohorts, the patients with poorer nutritional status were more likely to be older and have a high prevalence of CKD compared with normal nutrition. Those patients with severe malnutrition were less likely to be treated with angiotensin-converting enzyme inhibitors or angiotensin receptor blockers, beta-blockers, statins, and aspirin. More details of the baseline information of both cohorts are listed in Tables 1A,B.

**Table 1A:** Baseline characteristics of critical patients with AMI stratified by the CONUT score in the Chinese CIN database.

	**CIN**						**%, std.diff**		
**Characteristics**	**Overall (*n* = 2,075)**	**Absent (*n* = 429)**	**Mild (*n* = 985)**	**Moderate (*n* = 588)**	**Severe (*n* = 73)**	***P*-value**	**Absent vs. mild**	**Absent vs. moderate**	**Absent vs. severe**
**Demographic characteristics**									
Age, year	62.5 ± 12.3	55.6 ± 11.5	62.5 ± 11.9	67.0 ± 11.2	68.9 ± 11.6	<0.001	58.9	100.3	115.4
Female, *n* (%)	415 (20.0)	75 (17.5)	191 (19.4)	133 (22.6)	16 (21.9)	0.202	4.9	12.9	11.2
**Complication**									
CHF, *n* (%)	505 (24.3)	64 (14.9)	225 (22.8)	181 (30.8)	35 (48.0)	<0.001	20.4	38.5	76.1
CKD, *n* (%)	603 (29.1)	45 (10.5)	261 (26.5)	248 (42.2)	49 (67.1)	<0.001	42.1	77.1	142.8
Anemia, *n* (%)	859 (41.4)	69 (16.1)	380 (38.6)	352 (59.9)	58 (79.5)	<0.001	52.2	101.1	164.1
Hypertension, *n* (%)	1,049 (50.6)	194 (45.2)	479 (48.6)	333 (56.6)	43 (58.9)	0.001	6.8	23.0	27.6
Diabetes, *n* (%)	551 (26.6)	101 (23.5)	240 (24.4)	183 (31.1)	27 (37.0)	0.002	1.9	17.1	29.6
Stroke, *n* (%)	115 (5.5)	9 (2.1)	50 (5.1)	48 (8.2)	8 (11.0)	<0.001	16.1	27.8	36.5
STEMI, *n* (%)	1,842 (88.8)	383 (89.3)	886 (90.0)	512 (87.1)	61 (83.6)	0.16	2.2	6.8	16.7
PCI, *n* (%)	1,873 (90.3)	392 (91.4)	900 (91.4)	518 (88.1)	63 (86.3)	0.093	0.0	10.8	16.2
**Laboratory tests**									
Albumin, g/L	32.92 ± 4.45	37.53 ± 1.96	33.69 ± 2.90	29.43 ± 3.39	23.64 ± 4.01	<0.001	155.0	292.4	439.6
LYM, 10*^9^/L	1.46 (1.05, 1.96)	1.90 (1.58, 2.34)	1.51 (1.11, 1.98)	1.12 (0.81, 1.48)	0.79 (0.67, 1.10)	<0.001	60.5	126.8	187.7
Cholesterol, mmol/L	4.77 ± 1.22	5.55 ± 1.16	4.89 ± 1.08	4.18 ± 1.08	3.37 ± 1.03	<0.001	58.5	121.9	197.8
K^+^, mmol/L	3.80 ± 0.52	3.75 ± 0.41	3.74 ± 0.47	3.89 ± 0.63	3.99 ± 0.73	<0.001	2.4	25.2	40
Na^+^, mmol/L	137.62 ± 3.57	137.90 ± 3.02	137.71 ± 3.24	137.30 ± 4.10	137.44 ± 5.57	0.04	6.0	16.8	10.3
WBC, 10*^9^/L	12.08 ± 4.26	12.27 ± 3.65	12.01 ± 4.07	12.03 ± 4.63	12.18 ± 6.44	0.746	6.7	5.6	1.7
**Medications**									
ACEI or ARB, *n* (%)	1,263 (67.2)	296 (71.8)	636 (69.5)	304 (61.0)	27 (49.1)	<0.001	5.1	23.0	47.9
Beta blocker, *n* (%)	1,581 (84.1)	355 (86.2)	788 (86.1)	399 (80.1)	39 (70.9)	0.001	0.1	16.2	37.8
Statins, *n* (%)	1,788 (95.1)	397 (96.4)	881 (96.3)	462 (92.8)	48 (87.3)	0.001	0.4	15.9	33.6
Antiplatelet, *n* (%)	1,715 (91.2)	391 (94.9)	838 (91.6)	440 (88.4)	46 (83.6)	0.001	13.2	23.8	37
CCB, *n* (%)	170 (9.0)	39 (9.5)	76 (8.3)	44 (8.8)	11 (20.0)	0.033	4.1	2.2	30.1

*CHF, congestive heart failure; CKD, chronic kidney disease; STEMI, ST-segment elevation myocardial infarction; PCI, percutaneous coronary intervention; LYM, lymphocyte; K^+^, potassium ion; Na^+^, sodium ion; WBC, white blood cell; ACEI, angiotensin converting enzyme inhibitor; ARB, angiotensin receptor blocker; CCB, calcium blocker*.

**Table 1B:** Baseline characteristics of critical patients with AMI stratified by the CONUT score in the American MIMIC-III database.

	**MIMIC**						**%, Std.Diff**		
**Characteristics**	**Overall (*n* = 887)**	**Absent (*n* = 182)**	**Mild (*n* = 369)**	**Moderate (*n* = 271)**	**Severe (*n* = 65)**	***P*-value**	**Absent vs. mild**	**Absent vs. moderate**	**Absent vs. severe**
**Demographic characteristics**									
Age, year	70.1 ± 12.9	64.1 ± 13.5	70.6 ± 12.6	73.0 ± 12.0	71.9 ± 11.5	<0.001	49.7	69.5	62.2
Female, *n* (%)	332 (37.4)	67 (36.8)	134 (36.3)	101 (37.3)	30 (46.2)	0.505	1.0	0.9	19.0
**Complication**									
CHF, *n* (%)	428 (48.3)	62 (34.1)	176 (47.7)	156 (57.6)	34 (52.3)	<0.001	28.0	48.5	37.5
CKD, *n* (%)	447 (50.4)	51 (28.0)	179 (48.5)	172 (63.5)	45 (69.2)	<0.001	43.1	76.1	90.5
Anemia, *n* (%)	542 (61.1)	64 (35.2)	215 (58.3)	208 (76.8)	55 (84.6)	<0.001	47.6	92.3	116.9
Hypertension, *n* (%)	437 (49.3)	107 (58.8)	195 (52.9)	117 (43.2)	18 (27.7)	<0.001	12.0	31.6	66.1
Diabetes, *n* (%)	309 (34.8)	52 (28.6)	135 (36.6)	100 (36.9)	22 (33.9)	0.243	17.2	17.8	11.4
Stroke, *n* (%)	95 (10.7)	15 (8.2)	39 (10.6)	31 (11.4)	10 (15.4)	0.423	8.0	10.8	22.3
STEMI, *n* (%)	329 (37.1)	70 (38.5)	146 (39.6)	87 (32.1)	26 (40.0)	0.237	2.3	13.3	3.2
PCI, *n* (%)	292 (33.8)	65 (36.3)	126 (35.5)	82 (30.9)	19 (29.2)	0.469	1.7	11.4	15.1
Pre-AMI, *n* (%)	2 (0.2)	0 (0.0)	1 (0.3)	1 (0.4)	0 (0.0)	0.84	7.4	8.6	0.0
**Laboratory tests**									
Albumin, g/L	34.16 ± 5.74	39.13 ± 3.32	35.91 ± 3.82	30.76 ± 4.76	24.43 ± 3.46	<0.001	89.9	204.1	433.1
Lymphocyte, 10*^9^/L	1.37 (0.91, 2.06)	2.11 (1.72, 2.79)	1.39 (1.02, 2.04)	1.02 (0.72, 1.46)	0.78 (0.47, 1.11)	<0.001	44.7	68.2	81.5
Cholesterol, mmol/L	3.94 ± 1.25	5.04 ± 0.97	4.01 ± 1.05	3.37 ± 1.17	2.83 ± 0.94	<0.001	103.0	155.6	231.8
K^+^, mmol/L	4.26 ± 0.76	4.10 ± 0.64	4.26 ± 0.73	4.36 ± 0.86	4.29 ± 0.78	0.005	23.8	34.3	26.2
Na^+^, mmol/L	138.33 ± 4.18	138.81 ± 3.00	138.16 ± 4.08	138.44 ± 4.78	137.49 ± 4.69	0.117	18.3	9.3	33.6
WBC, 10*^9^/L	12.53 ± 6.06	12.33 ± 5.80	12.08 ± 5.57	12.91 ± 6.45	14.06 ± 7.43	0.058	4.5	9.5	25.8
**Medications**									
ACEI or ARB, *n* (%)	572 (64.5)	116 (63.7)	257 (69.7)	170 (62.7)	29 (44.6)	0.001	15.2	7.0	6.7
Beta blocker, *n* (%)	778 (87.7)	167 (91.8)	332 (90.0)	230 (84.9)	49 (75.4)	0.001	8.9	5.6	18.4
Statins, *n* (%)	726 (81.9)	158 (86.8)	315 (85.4)	207 (76.4)	46 (70.8)	0.001	19.9	18.3	1.7
Antiplatelet, *n* (%)	501 (56.5)	115 (63.2)	218 (59.1)	142 (52.4)	26 (40.0)	0.004	19.6	17.3	3.0
CCB, *n* (%)	189 (21.3)	22 (12.1)	89 (24.1)	62 (22.9)	16 (24.6)	0.008	5.2	8.1	32.4

*CHF, congestive heart failure; CKD, chronic kidney disease; STEMI, ST-segment elevation myocardial infarction; PCI, percutaneous coronary intervention; LYM, lymphocyte; K^+^, potassium ion; Na^+^, sodium ion; WBC, white blood cell; ACEI, angiotensin converting enzyme inhibitor; ARB, angiotensin receptor blocker; CCB, calcium blocker*.

### All-Cause Mortality

**Figure 2:**
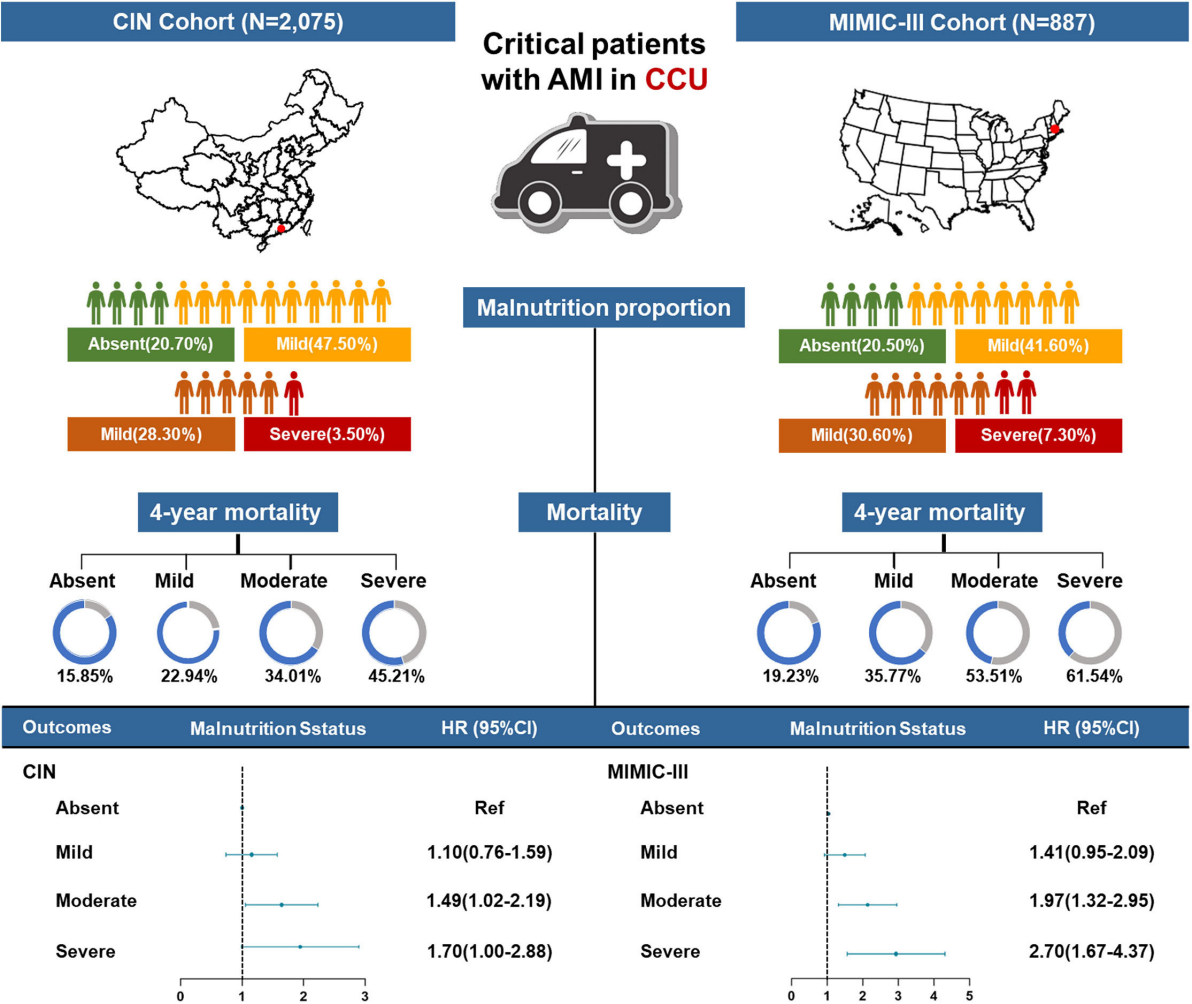
A summary graph of the role of malnutrition in critical patients with AMI in the CIN and MIMIC-III cohorts.

**Figure 3:**
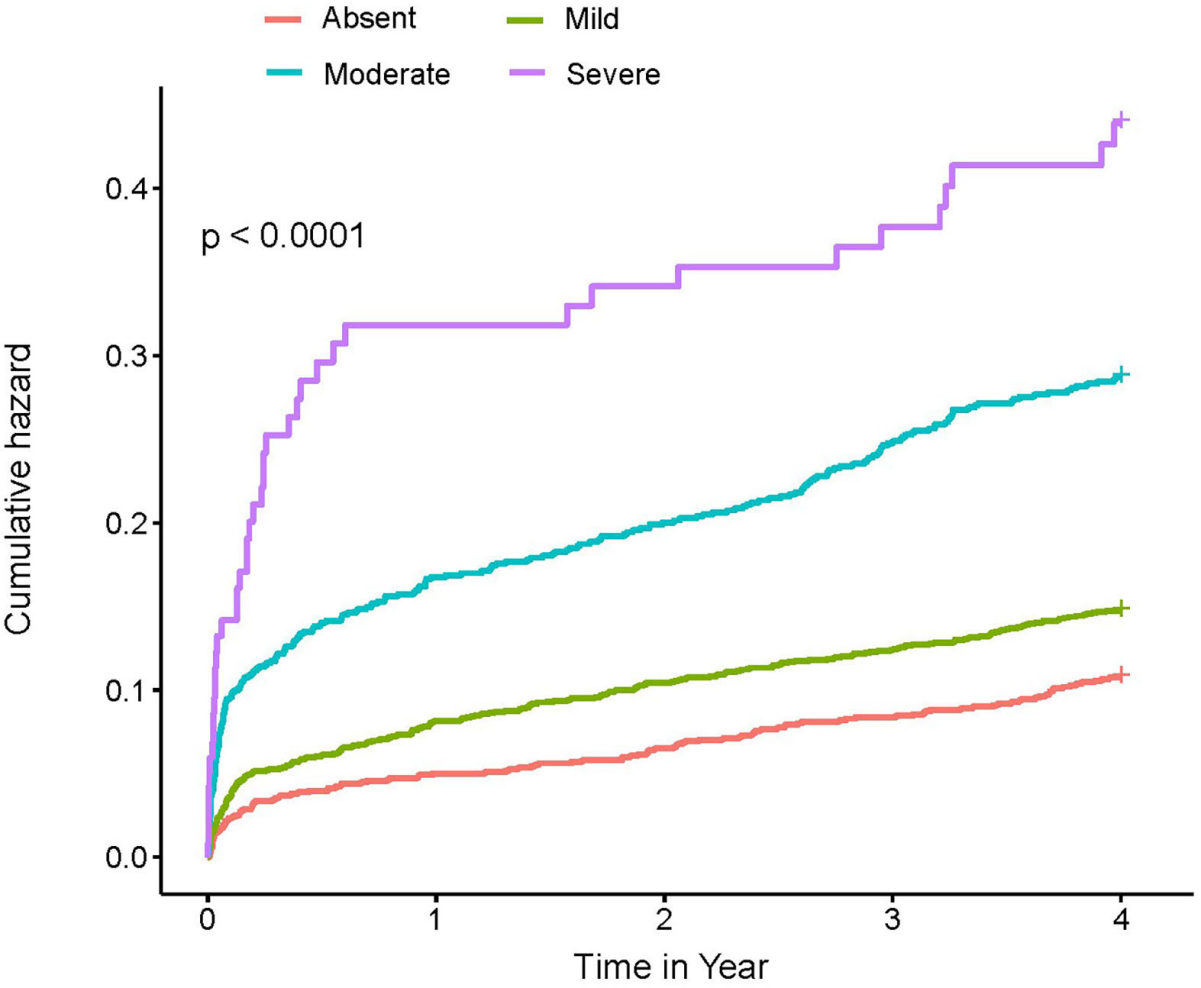
Kaplan–Meier curves of 4-year mortality among critical patients with AMI in the CIN cohort. A score of 0 to 1 is considered as absent; scores of 2–4, 5–8, and 9–12 reflect mild, moderate, and severe malnutrition, respectively. CONUT, Controlling Nutritional Status score.

**Figure 4:**
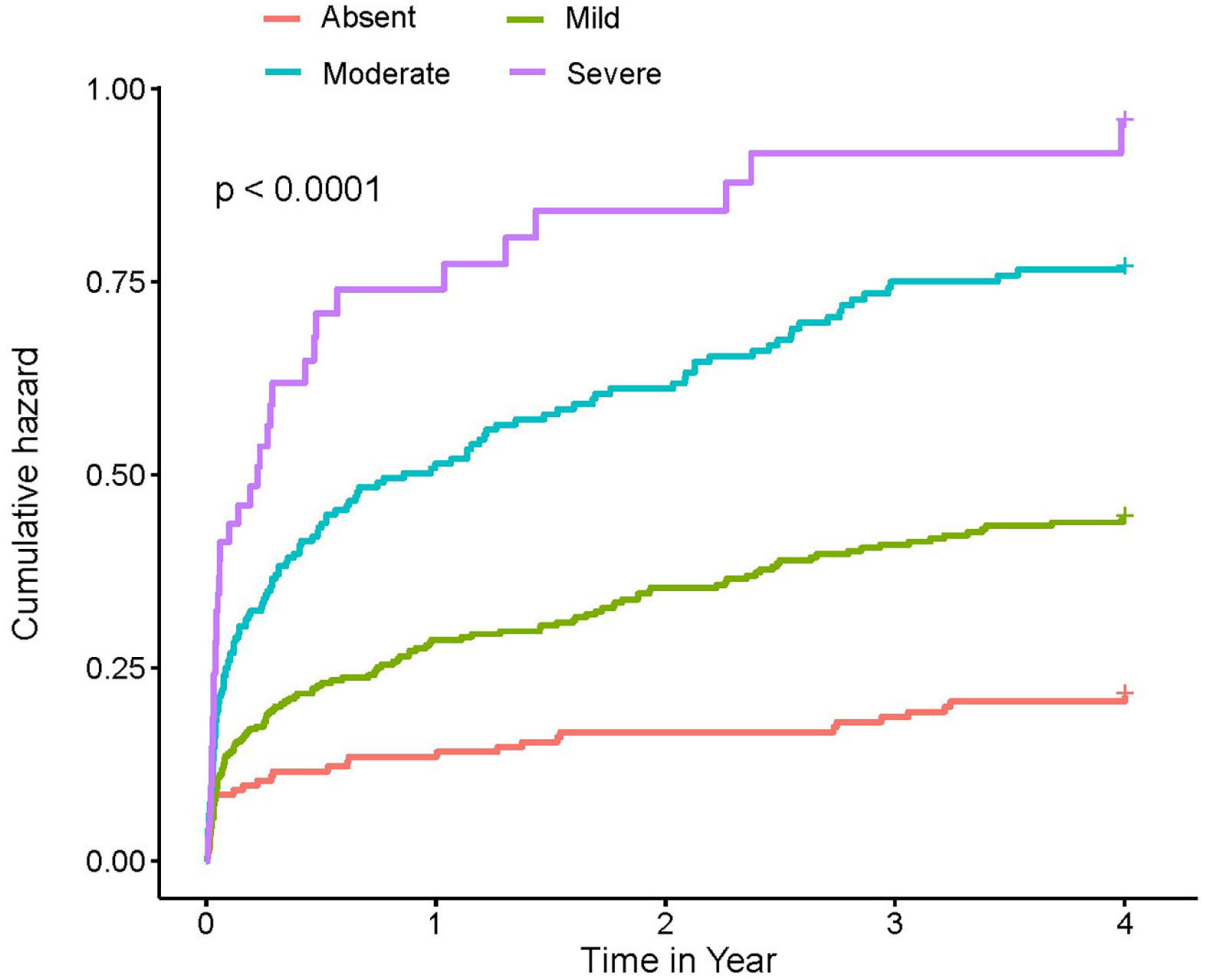
Kaplan–Meier curves of 4-year mortality among critical patients with AMI in MIMIC-III cohort. A score of 0 to 1 is considered as absent; scores of 2–4, 5–8, and 9–12 reflect mild, moderate, and severe malnutrition, respectively. CONUT, Controlling Nutritional Status score.

At a 4-year follow-up, the mortality of the four groups stratified by the CONUT score was 15.85, 22.94, 34.01, and 45.21% in the CIN cohort and 19.23, 35.77, 53.51, and 61.54% in the MIMIC-III cohort (Figure 2; Table 2). The Kaplan–Meier analysis demonstrated that worse nutritional status was associated with a higher incidence of all-cause mortality of critical patients with AMI in the two cohorts (Log-rank test, *p* < 0.0001, Figures 3, 4). Controlling for confounders, malnutrition was an independent risk factor for all-cause mortality [an adjusted hazard ratio for mild, moderate, and severe degrees of malnutrition, respectively: 1.10 (95% confidence interval (CI): 0.76–1.59), 1.49 (95% CI: 1.02–2.19) and 1.70 (95% CI: 1.00–2.88) in the CIN cohort and 1.41 (95% CI: 0.95–2.09), 1.97 (95% CI: 1.32–2.95), and 2.70 (95% CI: 1.67–4.37)] in the MIMIC-III cohort (Table 2). Similar relationships were shown in PSM and IPW analysis (Supplementary Figure 1 and Supplementary Table 2).

**Table 2:** Cox proportional hazards analyses of malnutrition to predict all-cause mortality in critical patients with AMI in Chinese CIN and American MIMIC-III databases.

			**Model 1**		**Model 2**		**Model 3**	
**Database**	**Group**	**Events, *n* (%)**	**HR (95% CI)**	***P*-value**	**HR (95% CI)**	***P*-value**	**HR (95% CI)**	***P*-value**
CIN	Absent	68 (15.9)	Ref		Ref		Ref	
	Mild	226 (22.9)	1.61 (1.13–2.30)	0.008	1.30 (0.91–1.87)	0.152	1.10 (0.76–1.59)	0.611
	Moderate	200 (34.0)	3.04 (2.14–4.33)	<0.001	2.16 (1.50–3.12)	<0.001	1.49 (1.02–2.19)	0.041
	Severe	33 (45.2)	5.23 (3.22–8.50)	<0.001	3.51 (2.13–5.79)	<0.001	1.70 (1.00–2.88)	0.049
MIMIC-III	Absent	35 (19.2)	Ref		Ref		Ref	
	Mild	132 (35.8)	2.03 (1.4–2.95)	<0.001	1.58 (1.09–2.31)	0.017	1.41 (0.95–2.09)	0.085
	Moderate	145 (53.5)	3.57 (2.47–5.17)	<0.001	2.52 (1.73–3.68)	<0.001	1.97 (1.32–2.95)	0.001
	Severe	40 (61.5)	4.75 (3.02–7.49)	<0.001	3.41 (2.15–5.38)	<0.001	2.70 (1.67–4.37)	<0.001

*Model 1: unadjusted*.

*Model 2: adjusted for age and sex*.

*Model 3: adjusted for age, gender, STEMI, chronic kidney disease, congestive heart failure, percutaneous coronaryintervention, anemia, stroke, hypertension, diabetes, sodium ion, and potassium ion*.

## Discussion

To our knowledge, this is the first study to examine the impact of malnutrition on all-cause mortality in critical patients with AMI from Chinese and American cohorts simultaneously. The main finding of the study is that the poor nutritional status, evaluated by the CONUT score, is widespread and predicts poor prognosis in critical patients with AMI either from CIN or MIMIC-III cohorts.

In our study, malnutrition is very common in the critical patients with AMI in both cohorts, with approximately one-third of the patients being at high risk for malnutrition. The prevalence of malnutrition was as high as 78% in a heterogeneous group of critically ill patients by validated nutrition assessment tools such as the Subjective Global Assessment (SGA) or Mini Nutritional Assessment (MNA) (12). However, Wada et al. also showed that one-fifth of patients were high-risk malnutrition based on the CONUT scores in a cohort of 2,092 patients who underwent PCI (13). In addition, Sergio Raposeiras Roubín et al. found that 11.2% of patients were moderate or severely undernourished by the CONUT score in a cohort of 5,062 patients with acute coronary syndrome (ACS) (11). In the studies of Sergio Raposeiras Roubín and Wada, the incidence of high-risk malnutrition was lower than in our study. The possible explanations could be as follows. The present study focused on the patients with severe myocardial infarction admitted to the CCU who probably have more comorbidities or poor basic conditions. In addition, the critical illness can affect overall energy requirements and caloric intake and losses, meanwhile, negatively affecting appetite and absorption (14).

The other finding of our study is that malnutrition was an independent risk factor in all-cause mortality in critical patients with AMI. There are studies that demonstrated the impact of nutritional status on the prognosis of patients with myocardial infarction or critically ill. Yue Shao et al. showed that undernutrition assessed by the nutritional indices [the Prognostic Nutritional Index score (PNI), CONUT, and the Geriatric Nutritional Risk Index (GNRI)] is strongly correlated with increased mortality rates of the patients in ICU (15). Sung HeeYooRN et al. showed undernutrition evaluated by GNRI at admission was an independent factor influencing in-hospital death in patients with AMI (16). Giuseppina Basta et al. found that the poor nutritional status evaluated by the CONUT score can influence the prognosis in elderly patients with STEMI (10). Few studies focus on the critical AMI in CCU. The risk of death for the patients with moderate-to-severe malnutrition increased significantly compared to the normal nutritional population in both databases. Noteworthy is the prevalence of high-risk malnutrition in patients with critical AMI in China seems lower, and the mortality is significantly higher in America. There are some reasons accounting for the phenomenon. The patients were older and had a higher prevalence of comorbidities and worse cardio-renal function in the MIMIC-III cohort. Moreover, PCI was an effective treatment for AMI, but the use of PCI was distinctly less than that in the CIN cohort. In addition, the enrollment period differs between the two study cohorts (American registry vs. Chinese registry, 2001–2012 vs. 2007–2018, respectively); the survival rate of the patients was improved with the continuous improvement of medical technology. The possible underlying mechanism linking malnutrition to all-cause mortality may be that the activation of inflammatory pathways plays an important role (17). Patients in the CCU are often in a state of heightened proinflammation, which leads to worse nutritional status. Theoretically, the activation of neurohormonal and inflammatory pathways that characterize cardiovascular disease may increase the catabolic demand, and patients with poor nutritional status may be more vulnerable to cardiac events (7).

All these findings strongly support the need for physicians to integrate in their daily practice the identification of malnutrition. Although malnutrition is often mentioned, it is rarely evaluated. Nutritional assessment in critically ill patients is not an easy task. Clinicians should stay abreast of the current scientific evidence to provide meaningful and effective nutrition guidance. One previous retrospective study, comprising 1,171 critically ill patients, showed that increasing caloric intake to 70% of resting energy expenditure was enough to lower mortalities (18). Nutritional therapy plays a fundamental role in the recovery of hospitalized patients. Some studies have shown that the use of targeted oral nutritional supplement strategies in older adults may reduce complications, mortality, and hospital readmissions (19). Nutritional intervention may prevent complications and improve the quality of life (20). Consequently, clinical trials are needed to prospectively evaluate the efficacy of nutritional assessments and interventions on outcomes in critical patients with AMI, and the optimal nutritional intervention therapy on the outcome in this population should be ensured.

## Study Limitations

This study has several limitations. First, due to the observational nature of this study, the results cannot be used to infer the direction of causality, and some potential covariates cannot be considered. Second, height and weight, which can reflect the nutritional status, were not available. Third, we did not apply different nutritional screening tools and compare their prognostic value of malnutrition for all-cause mortality. Unfortunately, information on educational attainment, marital status, socioeconomic, and characteristics was not available, which might help us understand the contributing causes of malnutrition. Finally, we did not reevaluate the time-dependent changes in nutritional status.

## Conclusion

Malnutrition is highly prevalent among critical patients with AMI and is independently associated with an increased risk of all-cause mortality. More research is required to propose adequate nutritional support to improve the nutritional status and prognosis in critical patients with AMI.

## Data Availability Statement

The original contributions presented in the study are included in the article/Supplementary Material, further inquiries can be directed to the corresponding authors.

## Ethics Statement

All traceable personal identifiers were removed from the analytic dataset to protect patients' privacy. The study protocol was approved by the Guangdong Provincial People's Hospital Ethics Committee and the study was performed according to the declaration of Helsinki.

## Author Contributions

SC, YF, and KC: conception and study design. JLu: execution. ZH, JW, XZ, YY, YP, KB, JX, JT, WC, BW, YK, JLi, YL, and KC: acquisition of data. ZH, JLu, and JW: analysis and interpretation. SC, LC, ZH, JLu, and YY: drafting and revising or critically reviewing the article. All authors contributed to important intellectual content during manuscript drafting or revision, accepts accountability for the overall work, agrees to be accountable for all aspects of the work, and approved the submitted version.

## Funding

This research was funded and supported by Fujian Province Natural Science Foundation (2018J01405, 2019J01617), Longyan City Science and Technology Plan Project (2015LY33), Startup Fund for Scientific Research by Fujian Medical University (2019QH1205), Beijing Lisheng Cardiovascular Health Foundation (LHJJ20141751), a study on the function and mechanism of the potential target for early warning of the cardiorenal syndrome after acute myocardial infarction based on transformism (DFJH201919), Natural Science Foundation of Guangdong Province General Project (2020A1515010940), and Guangdong Provincial Key Laboratory of Coronary Heart Disease Prevention (2017B030314041). The funders had no role in the study design, data collection, analysis, decision to publish, or preparation of the manuscript and the work was not funded by any industry sponsors.

## Conflict of Interest

The authors declare that the research was conducted in the absence of any commercial or financial relationships that could be construed as a potential conflict of interest.

## Publisher's Note

All claims expressed in this article are solely those of the authors and do not necessarily represent those of their affiliated organizations, or those of the publisher, the editors and the reviewers. Any product that may be evaluated in this article, or claim that may be made by its manufacturer, is not guaranteed or endorsed by the publisher.

## Abbreviations

AMI: acute myocardial infarction
CIN: Cardiorenal ImprovemeNt
MIMIC-III: Medical Information Mark for Intensive Care-III
CONUT: Controlling Nutritional Status
CI: confidence interval
CAG: coronary angiography
PCI: percutaneous coronary intervention
ICU: Intensive Care Unit
CCU: coronary care unit
ICD-9: Ninth Revision
SQL: structure query language
CHF: congestive heart failure
NYHA: New York Heart Association
STEMI: S-T segment elevation myocardial infarction
SGA: Subjective Global Assessment
MNA: Mini Nutritional Assessment
ACS: acute coronary syndrome
PNI: Prognostic Nutritional Index
GNRI: Geriatric Nutritional Risk Index.
