# Supplementary material for: Prevalence and Prognostic Impact of Malnutrition in Critical Patients With Acute Myocardial Infarction: Results From Chinese CIN Cohort and American MIMIC-III Database
Source: Front Nutr. 2022 Jun 15;9:890199. doi: 10.3389/fnut.2022.890199 (PMC9240700; doi:10.3389/fnut.2022.890199)
Supplement: Supplementary file 1 [file Table_1.DOCX]

Supplementary Material

# Supplementary Table 1 Malnutrition Screening Tools

|  |  | **Absent** | **Mild** | **Moderate** | **Severe** |
| --- | --- | --- | --- | --- | --- |
| **CONUT，points** |  | 0-1 | 2-4 | 5-8 | 9-12 |
| **Formula** | Serum albumin, g/dl | ≥3.50 | 3.00-3.49 | 2.50-2.99 | <2.50 |
|  | Albumin score | 0 | 2 | 4 | 6 |
|  | Total cholesterol, mg/dl | ≥180 | 140-179 | 100-139 | <100 |
|  | Cholesterol score | 0 | 1 | 2 | 3 |
|  | Lymphocytes, count/ml | ≥1600 | 1,200-1,599 | 800-1,199 | <800 |
|  | Lymphocytes score | 0 | 1 | 2 | 3 |

**Abbreviation:** CONUT: Controlling Nutritional Status score.

# Supplementary Figure 1 The intergroup differences of covariates with unmatched, matched and weighted adjustments in CIN and MIMIC databases

#
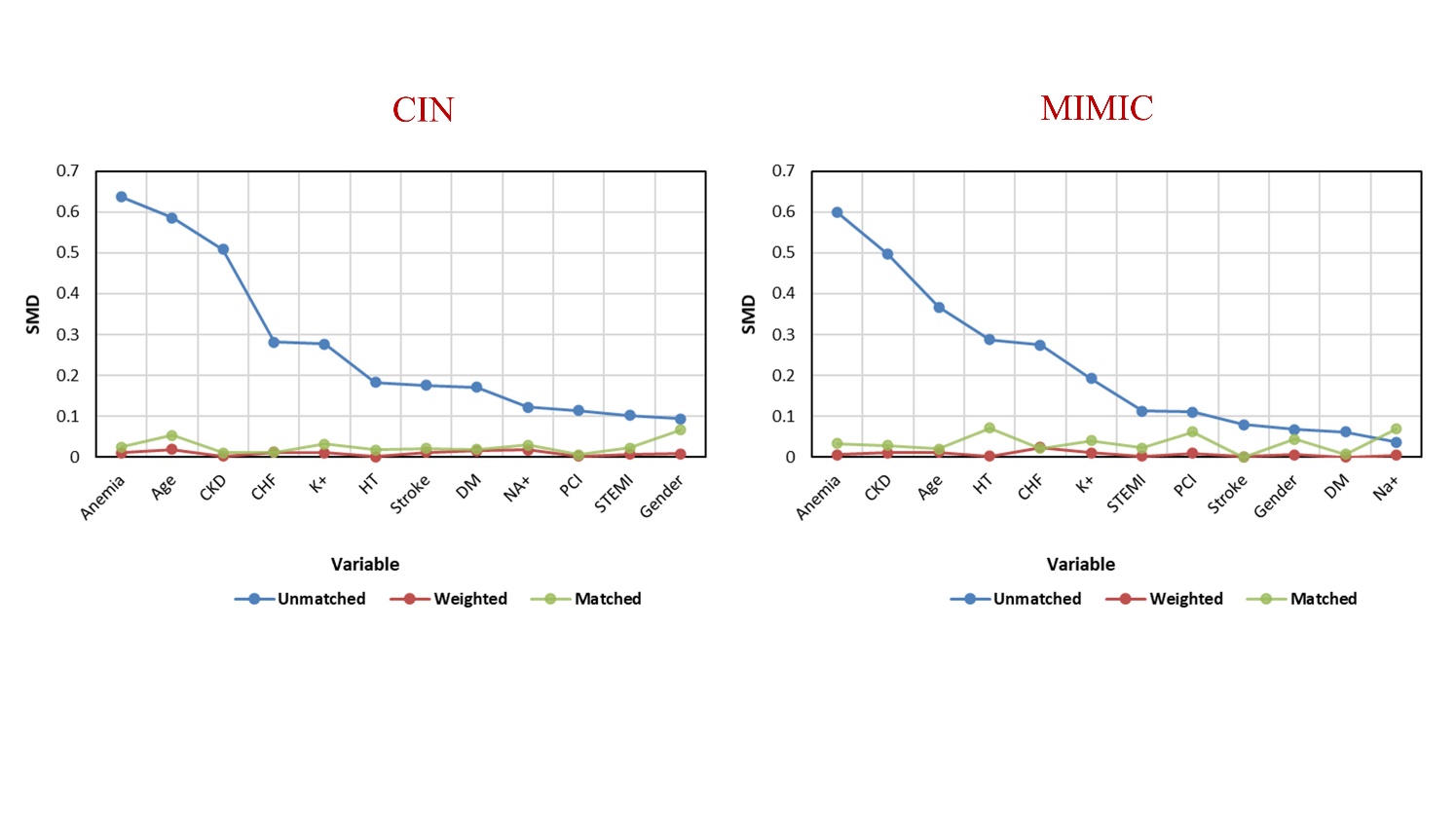


# Abbreviation: SMD: Standardized mean differences; CIN: Cardiorenal ImprovemeNt; MIMIC-III: Medical Information Mark for Intensive Care-III

# Notes: SMD of baseline confounding covariates between CONUT≤4 and CONUT>4 groups that was used to estimate the intergroup differences with unmatched (blue line), matched (green line) and weighted (red line) adjustments in CIN and MIMIC databases.

# Supplementary Table 2 Associations between malnutrition and all-cause mortality in the crude analysis and multivariable analysis using propensity-matched analysis and inverse probability weighting

| **Analysis** | **CIN** | |  | **MIMIC** | |
| --- | --- | --- | --- | --- | --- |
| **With matching** | | | | | |
| Crude analysis | Hazard ratio (95%) | P-value |  | Hazard ratio (95%) | P-value |
| Absent | Ref |  |  | Ref |  |
| Mild | 1.67(0.97-2.87) | 0.065 |  | 1.57(0.97-2.55) | 0.065 |
| Moderate | 1.65(0.96-2.83) | 0.071 |  | 2.07(1.29-3.32) | 0.003 |
| Severe | 3.64(1.91-6.94) | <0.001 |  | 2.88(1.67-4.96) | <0.001 |
| Multivariable analysis |  |  |  |  |  |
| Absent | Ref |  |  | Ref |  |
| Mild | 1.30(0.74-2.26) | 0.359 |  | 1.14(0.70-1.87) | 0.590 |
| Moderate | 1.45(0.84-2.50) | 0.187 |  | 1.60(0.99-2.58) | 0.055 |
| Severe | 2.13(1.09-4.15) | 0.026 |  | 2.31(1.33-4.02) | 0.003 |
| **With inverse probability weighting** |  |  |  |  |  |
| Crude analysis |  |  |  |  |  |
| Absent | Ref |  |  | Ref |  |
| Mild | 1.71(1.18-2.47) | 0.566 |  | 1.95(1.32-2.89) | 0.001 |
| Moderate | 2.02(1.38-2.96) | <0.001 |  | 2.39(1.59-3.58) | <0.001 |
| Severe | 3.69(2.13-6.39) | <0.001 |  | 3.28(1.97-5.45) | <0.001 |
| Multivariable analysis |  |  |  |  |  |
| Absent | Ref |  |  | Ref |  |
| Mild | 1.12(0.76-1.64) | 0.566 |  | 1.30(0.90-1.88) | 0.169 |
| Moderate | 1.54(1.03-2.31) | 0.036 |  | 1.85(1.27-2.71) | 0.001 |
| Severe | 1.83(1.04-3.25) | 0.037 |  | 2.31(1.42-3.75) | 0.001 |

**Notes:** We used propensity-matched analysis (PSM) and inverse probability weighting (IPW) adjustment to balance covariates between the CONUT score ≤4 and CONUT score > 4 groups in CIN and MIMIC databases.

Multivariable analysis adjusted for age, gender, STEMI, chronic kidney disease, congestive hearts failure, percutaneous coronary intervention, anemia, stroke, hypertension, diabetes, sodium ion and potassium ion.
